# Appetite-Controlling Endocrine Systems in Teleosts

**DOI:** 10.3389/fendo.2017.00073

**Published:** 2017-04-18

**Authors:** Ivar Rønnestad, Ana S. Gomes, Koji Murashita, Rita Angotzi, Elisabeth Jönsson, Hélène Volkoff

**Affiliations:** ^1^Department of Biology, University of Bergen, Bergen, Norway; ^2^Research Center for Aquaculture Systems, National Research Institute of Aquaculture, Japan Fisheries Research and Education Agency, Tamaki, Mie, Japan; ^3^Department of Biological and Environmental Sciences, University of Gothenburg, Gothenburg, Sweden; ^4^Departments of Biology and Biochemistry, Memorial University of Newfoundland, St John’s, NL, Canada

**Keywords:** appetite control, feed intake, hormones, neuropeptides, teleosts, adaptations, fasting, voracious feeding

## Abstract

Mammalian studies have shaped our understanding of the endocrine control of appetite and body weight in vertebrates and provided the basic vertebrate model that involves central (brain) and peripheral signaling pathways as well as environmental cues. The hypothalamus has a crucial function in the control of food intake, but other parts of the brain are also involved. The description of a range of key neuropeptides and hormones as well as more details of their specific roles in appetite control continues to be in progress. Endocrine signals are based on hormones that can be divided into two groups: those that induce (orexigenic), and those that inhibit (anorexigenic) appetite and food consumption. Peripheral signals originate in the gastrointestinal tract, liver, adipose tissue, and other tissues and reach the hypothalamus through both endocrine and neuroendocrine actions. While many mammalian-like endocrine appetite-controlling networks and mechanisms have been described for some key model teleosts, mainly zebrafish and goldfish, very little knowledge exists on these systems in fishes as a group. Fishes represent over 30,000 species, and there is a large variability in their ecological niches and habitats as well as life history adaptations, transitions between life stages and feeding behaviors. In the context of food intake and appetite control, common adaptations to extended periods of starvation or periods of abundant food availability are of particular interest. This review summarizes the recent findings on endocrine appetite-controlling systems in fish, highlights their impact on growth and survival, and discusses the perspectives in this research field to shed light on the intriguing adaptations that exist in fish and their underlying mechanisms.

## Introduction

**Figure 1:**
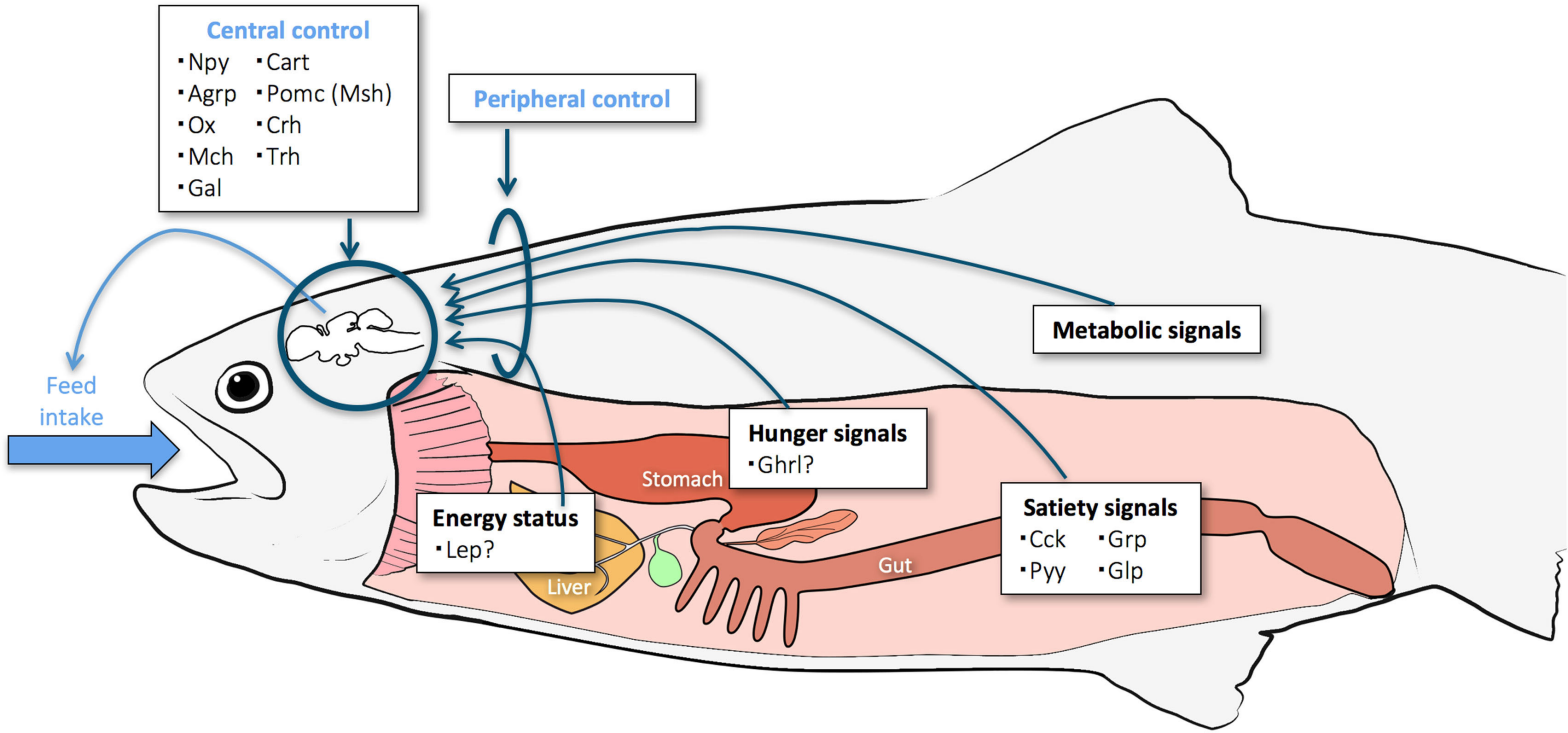
**Key organs and signaling pathways believed to be involved in control of appetite in fish**. Some of the central and peripheral endocrine factors explored so far are listed.

Control of food intake and energy metabolism is vital for the development and survival of an organism. These processes ensure optimal allocation of energy resources to cover the basic maintenance of metabolism and immune system, the cost of foraging and other daily activities, somatic growth, reproductive investment, and sufficient energy stores to survive periods of low food availability (1). Food intake is affected by external factors, such as temperature and photoperiod, stress, predators, and food availability, as well as by internal factors, such as genetics, life stage, gut filling, and stored energy. The hypothalamus is the hub that controls appetite and energy balance and integrates peripheral signals related to food intake and digestion, metabolism, and energy storage (Figure 1). These include not only endocrine signals (gut peptides, the focus of this review) but also other signals such as nutrient levels through central nutrient sensing systems and the presence/absence of food in the gastrointestinal (GI) tract through vagal afferents projecting to the brain.

Fishes represent over 30,000 species with an enormous variation in their ecological niches and habitats as well as life history adaptations, transitions between life stages and feeding behaviors. In the context of food intake and appetite control, common adaptations to extended periods of starvation or periods of abundant food availability are of particular interest. Also, the large variations in appetite between species and within a species (individual variation) are intriguing. A large fraction of fish species has indeterminate growth, i.e., these species continue to grow during their whole life span. This contrasts with growth in mammals and other model animals including zebrafish (*Danio rerio*), which reach a maximum length size as adults. Thus, while control of appetite and food intake is often viewed as a behavioral component of maintaining an energy balance (2), the general concept of energy homeostasis needs to be used with caution.

This review summarizes the recent findings on appetite-controlling systems in fish with a focus on peptide hormones. A major goal is to discuss perspectives in this research field that can reveal how fish adapt to their specific ecological requirements.

## Central Control

The physiological mechanisms that control appetite are relatively well conserved among vertebrates, and many of the neuropeptides and hormones involved in the central appetite regulation in mammals are also found in fish (3–7). However, differences in appetite-controlling systems can be found as a response to the large diversity in feeding habits of teleost species (8), yet the mechanisms for many of these adaptations remain unexplored.

Central signals arising in the hypothalamus are crucial for the control of food intake, and this brain area produces both orexigenic (appetite-stimulating) and anorexigenic (appetite-inhibiting) factors. The main hormones and neuropeptides so far described in teleosts and their possible involvement in the central control of appetite are presented in Figure 1 and described below.

### NPY

Neuropeptide Y (NPY) is one of the strongest orexigenic signals in mammals, and the NPY/agouti-related peptide (AgRP) neurons in the arcuate nucleus (ARC) are the principal inducer of feeding (9, 10). The relative importance of NPY in feeding regulation seems to vary among teleosts. In goldfish (*Carassius auratus*) (11, 12), carp (*Cyprinus carpio*) (13), channel catfish (*Ictalurus punctatus*) (14), zebrafish (15), rainbow trout (*Oncorhynchus mykiss*) (16), and Nile (17) and red (18) tilapias (*Oreochromis* sp.), NPY injections increase feeding. Food deprivation increases brain *npy* expression in several species, including goldfish (19), chinook and Coho salmon (*Oncorhynchus tshawytscha*; *Oncorhynchus kisutch*) (20), zebrafish (15, 21), winter skate (*Leucoraja ocellata*) (22), tiger puffer (*Takifugu rubripes*) (23), and winter (24) and Brazilian (25) flounder (*Pseudopleuronectes americanus*; *Paralichthys brasiliensis*), suggesting an orexigenic role. In some species, such as Atlantic cod (*Gadus morhua*) (26), tiger puffer (23), snakeskin gourami (*Trichogaster pectoralis*) (27), Brazilian flounder (25), channel catfish (28), and cobia (*Rachycentron canadum*) (29), *npy* brain expression levels are high around feeding time and decrease post-feeding, further suggesting a role of Npy as a short-term appetite stimulator in fish. Npy treatments have also been shown to stimulate fish growth/growth hormone (GH) secretion both *in vitro* [goldfish (30)] and *in vivo* [tilapia (*Oreochromis mossambicus*) (17, 31); orange-spotted grouper (*Epinephelus coioides*) (32)].

However, in Atlantic cod, fasting does not affect *npy* brain expression (26), in cunner (*Tautogolabrus adspersus*), short-term fasting decreases *npy* brain expression (33), and in both Atlantic salmon (*Salmo salar*) (34) and larval Atlantic halibut (*Hippoglossus hippoglossus*) (35), *npy* expression increases after feeding, suggesting that Npy might have a minor role as a feeding stimulator in these species. GH transgenesis, which results in increased feeding rates, does not affect brain *npy* levels in Coho salmon (36) and carp (37) but decreases *npy* levels in zebrafish (38).

In goldfish (39), Senegalese sole (*Solea senegalensis*) larvae (40), rainbow trout (41), and both Atlantic cod larvae (42) and adults (43), *npy* brain expression is modulated by diet, which is consistent with the role of NPY containing neurons in sensing the metabolic status (e.g., glucose levels) as reported for mammals (10) and fish [e.g., tilapia (44)]. However, in cobia (29), *npy* expression does not appear to correlate with diet-induced changes in food intake.

### CART

The peptide cocaine-amphetamine-related transcript (CART) was originally isolated from rat brain as a transcript regulated by acute administration of cocaine or amphetamine (45, 46). In goldfish, *cart* brain expression also increases following treatment with amphetamine (47). CART is a potent anorexigenic peptide in mammals (48–50) and birds (51), and CART injections inhibit food intake in goldfish (52).

Several *cart* genes have been identified in some fish species [e.g., two in goldfish (53), four in zebrafish (54), six in medaka (*Oryzias latipes*) (55), and seven in Senegalese sole (56)] whereas only one *cart* has been reported for others [e.g., Atlantic salmon (57), Atlantic cod (26), Atlantic halibut (35), and channel catfish (28)]. Post-feeding increases in *cart* brain expression have been reported for several fish species such as catfish (28), Atlantic salmon (34) and goldfish (53) suggesting that Cart acts as a short-term satiety factor in fish. Fasting has been shown to decrease *cart* brain expression in several fish species, and these changes are sometimes gene-specific. In goldfish, although the expression of both *cart* genes decreases after fasting, *cart1* appears more affected than *cart2* (53). In both zebrafish (58) and medaka (55), only one *cart* is affected by fasting, and in Senegalese sole, three out of seven *cart* genes are affected (56). However, fasting does not affect *cart* expression in other species such as winter flounder (24) or Atlantic halibut larvae (35), perhaps since only one gene has been identified in these species to date. Cart is also involved in sensing metabolic status, as hypothalamic *cart* mRNA levels change in response to changes in the levels of glucose in rainbow trout (41) or fatty acids in rainbow trout (59) and Senegalese sole (60).

### Orexin

Orexins (OXs) A and B (or hypocretin 1 and 2) are neuropeptide products of a single gene precursor, prepro-orexin (*pOX*), through proteolytic cleavage. Two orexin receptors have been identified, OXR1 and OXR2. OX-A binds to both receptors with similar potencies whereas OX-B binds preferentially to OXR2 receptors (61). In mammals, orexins have been implicated in the regulation of many physiological functions, including feeding, sleep–wake cycles, reproduction, and cardiovascular function (62–65). Orexins and/or orexin receptors have been identified in several fish species, including goldfish (66), cavefish (*Astyanax fasciatus mexicanus*) (67), zebrafish (68), barfin flounder (*Verasper moseri*) (69), orange-spotted grouper (70), Atlantic cod (71), winter flounder (72), and dourado (*Salminus brasiliensis*) (73). Orexins have been shown to increase feeding and locomotor behavior in both mammals (74) and fish (75–81). Consistent with its role as an orexigenic peptide, *ox* brain mRNA expression increases following fasting [e.g., cavefish (67), goldfish (77, 82), zebrafish (68), winter flounder (72), Atlantic cod (71), and dourado (73)] and around feeding time [e.g., cavefish (67), orange-spotted grouper (70), and goldfish (83)].

### Galanin

Galanin (GAL) is a 29–30 amino acid peptide first identified in mammals (84) and shown to have actions in brain and peripheral tissues to increase appetite and regulate metabolism (85, 86). Gal has been isolated in several fish species [reviewed in Ref. (87)] and appears to act as an orexigenic peptide. Injections of GAL stimulate food intake in goldfish (88) and tench (*Tinca tinca*) (89). Although long-term food deprivation does not affect brain *gal* mRNA expression in goldfish, the expression levels of *gal* decrease after the scheduled feeding time in fed fish, suggesting that Gal is a short-term regulator of appetite (90). Related to its role in metabolism, high *gal* mRNA expression has been linked to increased locomotion in zebrafish (91).

### MCH

Melanin-concentrating hormone (Mch) was first isolated from the salmon pituitary as a skin-paling factor (92, 93) and later isolated and identified as an orexigenic factor in mammals (94). In fish, the role of Mch in food intake regulation is still unclear. In goldfish, central injections of MCH inhibit appetite, and fasting induce a decrease in brain Mch-immunoreactive (ir) cells (95–97), suggesting an anorexigenic role. However, in other teleost species, such as winter flounder (98), barfin flounder (99), zebrafish (100), and Atlantic cod (101), fasting-induced increases *mch* mRNA levels and -ir cells, pointing to an orexigenic role.

### CRH

The corticotropin-releasing hormone (CRH) family includes CRH [or corticotropin-releasing factor (CRF)], urocortin (Ucn), urocortin 2, and urocortin 3. Members of the CRF family of neuropeptides have been shown to decrease feed intake in mammals (102). In goldfish, Crf and urotensin I (UI, the homolog of UCN in mammals) stimulate the hypothalamic–pituitary–interrenal axis (the fish homolog to the hypothalamic–pituitary–adrenal axis) to induce secretion of glucocorticoids (e.g., cortisol) and act as anorexigenic factors (6, 103). Central injections of CRF (104–106) or UI inhibit food intake in goldfish. Similar effects have been shown in rainbow trout (106). In Ya fish (*Schizothorax prenanti*), fasting decreases *crf* brain expression levels (107), consistent with the anorexigenic role of Crf-related peptides in fish.

### Melanocortin System

The vertebrate melanocortin system is phylogenetically well conserved, and it has been identified in fish, amphibians, and mammals (108–110). It consists of (1) melanocortin peptides, which includes melanocyte-stimulating hormones (α-, β-, and γ-MSH) and adrenocorticotropic hormone, all derived from the gene pro-opiomelancortin (*Pomc*), (2) five G protein-coupled melanocortin receptors (MCRs), and (3) endogenous melanocortin antagonists, agouti and AgRP (111). In vertebrates, components of the melanocortin system are involved in a diverse range of physiological functions, including regulation of food intake, appetite, and anticipatory behavior (112).

The melanocortins are posttranslational products of the POMC prohormone, which also gives rise to the opiate peptide β-endorphin. Posttranslational processing of the POMC prohormone is tissue-specific, which results in the production of different POMC peptides by different cell types and, therefore, multiple physiological functions. *Pomc* is a single copy gene in mammals and birds, but in most teleosts, there are two to three different *pomc* transcripts [e.g., zebrafish (113), carp (114), barfin flounder (115), gilthead sea bream (*Sparus aurata*) (116), and sockeye salmon (*Oncorhynchus nerka*) (117)], proposed to result from the whole or partial genome duplication (118). In salmonids, Atlantic salmon and rainbow trout, three copies of *pomc* gene and one splice variant have been described, i.e., *pomc* (*-a1, -a2, -a2s*, and *-b*) (119, 120). However, the functions of the fish *pomc* subtypes remain largely unexplored. In rainbow trout, fasting induces increased expression levels of both hypothalamic *pomca1* and *pomcb* (121), whereas in olive flounder (*Paralichthys olivaceus*), *pomc2* but not *pomc1* and *pomc3* mRNA levels increase with fasting (122), suggesting a form-specific response of *pomc* in some species.

The repertoire of MCRs (MC1R to MC5R) found at the target cells has undergone significant diversification and specialization. Therefore, MCRs differ in their affinity for the different melanocortins, agouti, and AgRP. Of importance to energy homeostasis are MC3R and MC4R that are expressed throughout the central nervous system (CNS). Fish Mcr and ligands are expressed in a highly conserved pattern relative to mammals (123, 124). This conservation is also seen in the melanocortin neural circuits involved in hypothalamic control of energy homeostasis, underlining that the melanocortin functions originated early in evolution (125). The presence of Mc4r in teleosts has been reported in several species [e.g., goldfish (126), zebrafish (127), spotted scat (*Scatophagus argus*) (128), snakeskin gourami (129), fugu (109), common carp (130), and Ya fish (131)]. In Atlantic salmon, several paralogs of Mcr have been described, *mc1r* (*-p1* and *-p2*), *mc2r, mc4r* (-*a-p1, -a-p2, -b-p1*, and *b-p2*), *mc5r, mrap2* (*-p1* and *-p2*) (Lars Ebbesson, Uni Environment, Bergen, Norway, personal communication). Mc3r seems to have been lost early in teleost evolution and is not present in salmonids, as observed for pufferfishes, tiger puffer and tetraodon (*Tetraodon nigroviridis*) (132). The only known *mc3r* in teleosts is the zebrafish *mc3r*; however, *mc3r* has also been identified in the spiny dogfish (*Squalus acanthias*) (133). In snakeskin gourami, the *mc4r* mRNA expression varies during daily feeding and fasting period, and its correlation with *npy* expression indicates a role in feed intake control (27, 129). However, in barfin flounder and sea bass (*Dicentrarchus labrax*), progressive fasting did not modify the hypothalamic *mc4r* mRNA expression (134, 135). Intracerebroventricular injections of MCR agonist decrease food intake in juvenile rainbow trout (136) and in goldfish (126, 137) in a dose-dependent manner, whereas the injection of MCR antagonists increases food intake in rainbow trout and in goldfish (137). The importance of Mc4r in the regulation of fish growth is also emphasized by naturally occurring mutations of the Mc4r in swordtails (*Xiphophorus nigrensis* and *Xiphophorus multilineatus*), which dramatically affects growth (138, 139).

An interesting fact is the existence of two endogenous antagonists in the melanocortin system, agouti and AgRP. These proteins are paracrine-signaling molecules and act as subtype-selective endogenous antagonists. AgRP exerts its major physiological function in the hypothalamus, where it acts as a potent orexigenic factor (140) due to its ability to antagonize the MC3R and MC4R (141). *agrp* genes have been identified in several fish species (57, 124, 126, 130, 131, 142–144). Hypothalamic *agrp* expression in goldfish (137), sea bass (*agrp1*, not *agrp2*) (144), and zebrafish (124) dramatically increased during fasting. In addition, GH-transgenic common carp has higher feed intake and higher hypothalamic *agrp1* mRNA expression levels than non-transgenic fish (37). *agrp* mRNA abundance in the hypothalamus of rainbow trout (59) and Senegalese sole (60) also responds to changes in the levels of specific fatty acids. Altogether, it is suggested that the role of AgRP in energy homeostasis and its relation to the melanocortin system is conserved across vertebrates (51, 145).

## Anatomical Locations of Central Appetite Control Systems

Control of appetite is an evolutionarily conserved process resulting from a close interplay between multiple neuronal and peripheral signals, which are integrated in the hypothalamus and processed in a specific spatial and temporal order to regulate hunger and satiety (4, 146). The mammalian hypothalamus consists of numerous interconnecting nuclei organized into complex neuronal networks where ARC nucleus, ventromedial nucleus (VMN), dorsomedial nucleus (DMN), paraventricular nucleus (PVN), and lateral hypothalamus (LH) play crucial roles in food intake control and energy expenditure [reviewed in Ref. (146)]. The ARC contains two distinct neuronal populations referred to as “first order” neurons, releasing appetite stimulators NPY/AgRP and appetite suppressors POMC/CART (1, 147). Neuronal projections from the first order neurons connect to other hypothalamic nuclei (PVN, DMN, VMN, and LH) (148). These “second order” nuclei express potent orexigenic factors such as orexins and MCH in the LH, and anorexigenic neuropeptides such as CRH and thyrotropin-releasing hormone (TRH) in the PVN. Lesioning studies in these nuclei have long recognized their functional significance in generating satiety and hunger responses [reviewed in Ref. (149)].

The existence of a functional (and to lesser extent anatomical) equivalence of appetite-controlling brain regions in fish has been demonstrated, based on electrical stimulation and brain lesion studies [reviewed in Ref. (4)]. The teleostean hypothalamic neurons are organized in a similar fashion as their mammalian counterparts and are distributed in conserved clusters within the ventral diencephalon (150–153). Yet, very little is known about the fish anatomical homologs to mammalian hypothalamic VMN, DMN, PVN, and LH nuclei, owing to the lack of specific neuronal molecular markers for distinct neuronal classes. In addition, expression domains of fish appetite control genes do not appear anatomically confined to their putative hypothalamic homologous areas.

The lateral tuberal nucleus (NLT; also known as ventral periventricular hypothalamus Hv) might be a feeding center and the teleostean homolog of the mammalian ARC [reviewed in Ref. (153)]. *pomc, agrp*, and *leptin* receptor transcripts are found in neurons within the NLT of goldfish (126, 137) and zebrafish (154), and ir and/or gene expression studies have identified Npy in the NLT of several teleosts (155, 156), as well as sturgeon [*Acipenser transmontanus* (157) and elasmobranch fish (158)]. *npy* and *cart* transcripts are also present in the NLT of juvenile Atlantic cod (159). In addition, Msh-α and Agrp-ir-cells are found in discrete populations in the NLT of zebrafish (125).

A recent study shows high homology between the zebrafish neurosecretory preoptic area (POA) and the mammalian PVN (153, 160). This homology is consistent with the presence of fish *trh* and *crh* ortholog genes in the POA, although their expression is not exclusive to the POA (161–164). The mammalian PVN is an important site of NPY synthesis and release (146, 165), and recent evidence indicates that Npy-ir cells and *npy* mRNAs are also present in the POA of fish (159, 166), further supporting functional homology between PVN and POA structures.

Functional and to some extent anatomical homologies could also exist between the mammalian and fish LH. In mammals, LH is an important site of orexins and MCH expression and believed to act as a “feeding center” (146). The LH is the site of transit for neuronal fibers interconnecting hypothalamic nuclei and forebrain to midbrain structures. A similar neuronal pattern has been observed in the LH of zebrafish, where *pOx*-expressing neurons send projections to the midbrain and the spinal cord (167, 168). In addition to the LH, the POA and the rostral NLT are also important sites of *pOx* expression in fish, as recently observed by double-fluorescence *in situ* hybridization in Atlantic cod larvae, in which the caudal domain of *pOx*-expressing neurons in the POA overlaps with the rostral-most *cart* cell population in the NLT (159). *pOx* mRNA expression in the POA has also been reported in zebrafish (169).

Furthermore, the strong expression of *cart* mRNAs and the absence of orexigenic modulators such as *npy* or *pOx* in the diffuse nucleus of the inferior hypothalamic lobe of Atlantic cod has recently led to the hypothesis that this nucleus may be the VMN homolog and that may serve as “satiety center” in fish (159) as in mammals (149, 167).

mRNAs of several appetite signals have been detected in the brain of different fish in extra-hypothalamic areas analogous to those characterized in mammals, suggesting a functional relationship between them (26, 41, 58, 159, 170, 171). It is, however, important to underline that canonical appetite genes (e.g., *Npy* and *Cart*) in mammals are modulated by many factors and their wide brain distribution may reflect various physiological roles and responses to changing environmental conditions (45, 172). All these mechanisms are still largely unknown in fish.

## Peripheral Signals

### The GI-Tract

The GI-tract is the largest endocrine organ in vertebrates and produces around 30 different neuropeptides and hormones. These peptides act on several tissues, including the GI-tract itself, exocrine glands, and the CNS (173, 174). Most of the GI peptides are sensitive to the gut nutrient content, and some of them are important in the control of appetite and meal size (174, 175). GI peptides may act on the CNS *via* an endocrine action by traveling in the blood, which requires that they pass the blood–brain barrier, and/or by stimulating afferent vagal nerve fibers (174, 176, 177). Studies on rainbow trout show that appetite returns when 80–90% of the stomach content has been emptied (178), indicating that gut filling, feed digestion, and transit rates may affect appetite control with both hunger and satiety signals. Indeed, most of the gut-derived appetite-regulating factors are also involved in digestion, thus coordinating these two processes (179).

#### GHRL

Ghrelin (GHRL) is mainly produced in the stomach of fish and mammals, or in the intestine of some stomachless species (180). Ghrl has been shown to have an orexigenic function in several fish species, including goldfish (177, 181), tilapia (182), brown trout (*Salmo trutta*) (183), and grass carp (*Ctenopharyngodon idellus*) (184), which is consistent with its role in mammals (185, 186). However, in rainbow trout, opposite effects of Ghrl on feed intake have been reported from two independent studies: one showed that central injection of Ghrl increased feed intake after 24 h (187) whereas the other study showed that short-term (1 h) central and long-term (weeks) peripheral administration of Ghrl suppressed appetite (174). The different time scales may, at least partly, explain the contradictory results. Recently, an anorexigenic response was also reported in channel catfish after Ghrl administration (188). In goldfish, appetite-regulating neuropeptides in the CNS, such as Npy and Ox, seem to mediate Ghrl-induced feeding (181, 189), but interactions between Ghrl and central appetite regulators are inconsistent in other examined fish species. For example, Ghrl increased (in tilapia and rainbow trout) (182, 187), decreased (in rainbow trout) (190), or did not affect (in brown trout and channel catfish) (183, 188) hypothalamic *npy* expression. Moreover, Ghrl decreased (in rainbow trout) (187) or had no effect (in channel catfish) (188) on *pomc* expression. A CRH receptor antagonist (α-helical CRF 9–41) abolished Ghrl-induced feeding (191) whereas Ghrl administration did not affect central *crh* expression in rainbow trout (187). In goldfish, it appears that peripheral Ghrl may stimulate feeding by acting on gastric vagal afferents that transmit information to brain appetite centers (177). Indirect effects on food intake, through stimulatory actions on digestion, could subsequently affect onset of feeding. For instance, rat GHRL evoked intestinal contraction in zebrafish (192, 193), but homologous Ghrl did not affect GI-tract contractility in goldfish and rainbow trout (194). The presence of GH secretagogue receptor in the fish pituitary and brain (particularly hypothalamus and telencephalon) also suggests a direct action of octanoylated Ghrl in these tissues (195, 196).

#### CCK

Cholecystokinin (CCK) is secreted by the proximal intestine and mainly acts as a short-term satiety factor at the same time as it promotes digestion through its many actions on the digestive system of vertebrates (174, 197). CCK is characterized by an evolutionary conserved biologically active C-terminal octapeptide (CCK-8) among vertebrates (198, 199), and Cck-ir cells have been observed in the intestine of most fish groups (174). Central or peripheral administration of sulfated CCK-8 suppresses food intake in goldfish (200) and channel catfish (14). Oral CCK administration inhibits feed intake in sea bass (201), while oral treatment with CCK antagonists increases food intake in rainbow trout (202). A single *cck* gene has been cloned in several teleost species, including yellowtail (*Seriola quinqueradiata*) (203), Atlantic herring (*Clupea harengus*) (204), and pirapitinga (*Piaractus brachypomus*) (205). However, two different *cck* sequences were identified in Japanese flounder (*Paralichthys olivaceus*), tetraodon (206), Atlantic salmon (207), and white sea bream (*Diplodus sargus*) (208), and three distinct *cck* genes exists in rainbow trout (209). All the identified *cck* genes in teleosts are predominantly expressed in the GI-tract and brain, including hypothalamus, telencephalon, and optic tectum.

Both circulating levels of Cck and *cck* gene expression are influenced by macronutrients, although these effects appear to be species-specific. For example, rainbow trout fed a high fat diet had higher plasma Cck levels compared with fish fed a high protein diet (210) and oral administration of single bolus of fat (oleic acid) or protein (casein), but not carbohydrate (starch), increased *cck* expression in yellowtail gut (211). In addition, *cck* expression levels increased following a meal in yellowtail pyloric caeca (212) and circulating Cck levels increase postprandially in rainbow trout (213). Fasting decreases gene expression or protein levels of Cck in the gut of yellowtail and white sea bream (203, 208). These results support the anorexigenic function of Cck and the conservation of this function in the teleost lineage. Some studies, however, show opposite effects; in Coho salmon, *cck* gene expression in the gut increased during winter fasting (214). In Atlantic salmon, on the other hand, intestinal *cck* mRNA expression was unchanged after 6 days of fasting (207). Furthermore, there are variations in the distribution pattern of Cck-producing cells within the intestinal segments among species (204, 215, 216) as well as in the fasting response among *cck* isoforms (207–209) suggesting diverging roles among species and *cck* isoforms. The action of CCK is initiated by its binding to two subtypes of cognate receptors (CCK-1R and CCK-2R), which results in satiety (197). Cck receptor genes have been isolated in yellowtail (*cck-1r*) (217), Atlantic salmon (*cck-1r, cck-2r1*, and *cck-2r2*) (218), and goldfish (*cck-1r* and *cck-2r*) (219). The primary structure of fish Cck receptors as well as their tissue distribution patterns is highly conserved; *cck-1r* is widely distributed within the GI-tract, while *cck-2r* is mainly expressed in the brain. Furthermore, *cck-1r* expression levels increased after feeding in yellowtail pyloric caeca (217), suggesting that Cck-1r mediates the effects of Cck on appetite, as in mammals (220). Further studies on Cck receptors are required to elucidate the detailed mechanisms underlying the anorexigenic function of Cck in fish.

#### PYY

Peptide YY (PYY) is a member of the NPY family. But, while NPY is well known to have a strong orexigenic function in the CNS (1), peripheral PYY mainly produced in the distal intestine (221) inhibits food intake in mammals (222). PYY consists of two forms: 36 (PYY1–36) or 34 (PYY3–36) amino acids (223). Two isoforms of the gene *pyy, pyya*, and *pyyb* (previously named *py*) (224) have been identified in teleost species, including sea bass (155), Atlantic salmon (207), and piranha (*Pygocentrus nattereri*) (225). To date, the *pyy* gene expression patterns are similar among the studied fish species, being predominantly expressed in the brain and GI-tract (203, 226). On the other hand, controversial results have been reported when analyzing intestinal segments from fed versus fasted fish. Fasting decreased (in piranha) (225), increased (in yellowtail) (203), or did not affect (in Atlantic salmon) (207) *pyy* expression. After feeding, GI-tract *pyy* mRNA expression increased in grass carp (227), while it decreased in yellowtail (212). These observations suggest that *pyy* response to fasting/feeding might be species-specific (225). Central and peripheral Pyy1–36 injection reduced food intake in goldfish (228), while administration of the truncated form Pyy3–36 had no effect on food intake in channel catfish (188) or goldfish (228). These results suggest that Pyy3–36 is not a major endogenous form of Pyy in fish (228, 229). The current mammalian model indicates that PYY suppresses appetite through the inhibition of NPY and subsequent activation of POMC neurons (230); however, the effects of GI-tract-derived Pyy on CNS are still uncertain in fish. PYY inhibits GI motility and pancreatic exocrine activity in mammals (175), and a similar digestive function has also been suggested for Pyy in teleosts (207, 211).

#### GRP

Gastrin-releasing peptide (GRP) is a homolog of the amphibian bombesin (Bbs) and is released from the GI-tract. In mammals, GRP decreases feed intake (231) and stimulates gastric acid secretion and motility (232). Bbs/Grp also appears to stimulate gastric secretion and motility in teleosts (233–235). In teleost species, Bbs/Grp-like peptides have been detected in the GI-tract of rainbow trout (236) and chub (*Squalius cephalus*) (237), and *bbs*/*grp* cDNA sequences have been published for goldfish (238), zebrafish (239), and Atlantic cod (240). Restricted feeding decreased *grp* expression in the gut of Atlantic cod (240) and zebrafish, but the *grp* decreasing pattern was reversed in the latter after refeeding (239). Central or peripheral injections of Bbs suppress feed intake in goldfish (200), which might be attributed to Bbs-induced reduction in *ghrl* gut expression (241). In addition, peripheral injections of Bbs/Grp decrease feeding in channel catfish (188) and Coho salmon (242). On the other hand, feeding status or diet composition does not seem to influence plasma Grp levels in rainbow trout (210). These observations indicate that teleost peripheral (gut) Grp may have an anorexigenic function and its signaling pathway is not endocrine but *via* neuronal circuits or local paracrine action, as proposed for the mammalian model (231).

#### The Evolution of Leptin Teleost Genes

The leptin gene (O*b*) was first identified in double mutant (*Ob/Ob*) mice (243) and presented an obese phenotype associated with impaired metabolic functions. Since obesity is linked to several comorbidities in humans, including type II diabetes and cardiovascular disease (244, 245), leptin has been extensively investigated in both humans and murine models. The first fish leptin was identified in 2005 (246). Leptin orthologs and several duplicated paralogs, originating from the whole-genome duplication (WGD) events, have recently been identified in teleost species (247, 248). These include 3R-leptin duplicated paralogs (A and B) in zebrafish (249), medaka (250), orange-spotted grouper (251), tilapia (252), chub mackerel [*Scomber japonicas* (253)], and European and Japanese eel [*Anguilla anguilla* and *Anguilla japonica* (254)], as well as two conserved leptin paralogs [*lepAI*/*lepAII* and *lepA1*/*lepA2* (255, 256)]; in common carp and goldfish, as a result of the ancestral lepA doubling at the basal root of cyprinids (256, 257) about 8 million years ago (258). In salmonids, additional “recent” 4R-leptin duplicates have been identified consistently with the (pseudo) tetraploid state of their genome (259–261).

Leptin functions are mediated *via* class-I helical cytokine receptors (long-form LEPR) through intracellular JAK/STAT signal transduction pathways (262, 263), in an evolutionarily conserved manner as suggested by transfection assay studies for carp (264), rainbow trout (265), and tilapia (252) receptors. In humans, alternative splicing of the LEPR gene leads to expression of long (LEPRb) and short (LEPRa, -Rc, -Rd) isoforms (266).

Single leptin receptors have been identified in most fishes (154, 250, 251, 267, 268), but two 3R-duplicated *lepR* genes are present in the ancestral teleost eel. This suggests that a loss of the second *lepR* (*lepRB*) may have occurred after the clupeocephals/elopomorphs split during teleost radiation (254). At the root of extant salmonids, the *lepRA* was then further duplicated by the 4R-WGD as deduced by the recently cloned *lepRA2* in Atlantic salmon (269). Like mammals, LepR isoforms that arise from alternative splicing of the C-terminal exon have been identified in fish (260, 264, 270, 271). LepR splice variants encode for circulating soluble binding proteins (LepBPs) that may function in leptin modulation, transport, and clearance (265, 271, 272). The characterization of the *leptin*-*lepR* system in the context of WGD(s) in teleost genomes and overall evaluation of their functional significance are instrumental to understand to which extent leptin duplicates have contributed to species-specific feeding adaptations.

#### Leptin Signaling—The Liver and Adipose Tissue

In mammals, leptin is an anorexigenic hormone released into the blood stream mainly by adipocytes. It acts as a lipostatic factor in a negative feedback loop between fat tissue and hypothalamic brain regions so that the organism can maintain energy balance and adequate fat mass reservoirs (273–276). Leptin signaling in the CNS is exerted on different hypothalamic neurons to inhibit the expression of the orexigenic NPY and AgRP and stimulate anorexigenic POMC and CART (120, 277–280). In fish, liver is the main secretory source of LepA (249, 250, 260, 270, 281–283), although some studies reported moderate mRNA expression and secretion from the adipose tissue (260, 270, 281, 284, 285). Central and peripheral administration of recombinant leptin, using homologous or heterologous leptin, produces anorectic effects in several fish species, suggesting that the regulatory role of leptin on appetite is well conserved in vertebrates (120, 279, 282, 286–289).

Leptin variations in response to feeding status (postprandial, short- and long-term fasting/food restriction) have been reported at the level of gene expression and protein among fish orthologs as well as among paralogs. For instance, postprandial increases in hepatic *lepA* and *lepB* expression are observed within 9 h in common carp (255), and hepatic *lepA* in orange-spotted grouper (251) and mandarin fish [*Siniperca chuatsi* (289)], suggesting that leptins may act as a satiety signal. In longer-term fasting (after 7 days and after 3 weeks), a significant increase in hepatic *lepA* expression was observed in orange-spotted grouper, but not in carp (289). Prolonged feed restriction induced hepatic upregulation of *lepA* expression in salmonids (290–292) and chub mackerel (253). In contrast, liver *lepA* expression decreases during catabolic states in striped bass (*Morone saxatilis*) (282), and hepatic mRNA expression of *lep1, lep2, lepRa*, and *lepRb* does not correlate to feeding status in eels (254).

*lepB* expression is low or absent in the liver of several teleosts and is mostly found in the CNS (253, 261, 289). The brain expression profiling of *lepA-B* paralogs in relation to feeding status shows species-specific variations among orthologs, paralogs, and time exposure to catabolic states. For instance, short-term fasting induces a downregulation of both *lepA* and l*epB* in the brain of mandarin fish (289), whereas it has no effect on *leptin(s)*/*lepR* in orange-spotted grouper (251). Long-term fasting has no effect on either *lepA* or *lepB* in Nile tilapia, *Oreochromis niloticus* (252), and eel (254), while in salmon, it induces upregulation of *lepA1* and *leprA1* expression and downregulation of *lepB1–2* genes in the brain (269). The increases in *lepA1* and *leprA1* mRNA upon fasting are in line with most studies on plasma leptin in salmonids (291–293). Also, in Mozambique tilapia (*Oreochromis mossambicus*), hepatic *lepA* mRNA as well as circulating LepA is higher in fasted than fed fish (294), as is seen with salmonids. Rising leptin plasma levels could be adaptive during catabolic states inducing anorexigenic effects at the level of the CNS, and a consequent reduction of energy-demanding foraging behavior during periods of limited food availability (291, 295). Interestingly, in burbot (*Lota lota*), plasma leptin levels decrease following fasting at 2°C but not at 10°C, implying that metabolic rate may influence leptin in catabolic conditions (296).

Given the lipostatic role of leptin in mammals, putative similar roles have been investigated in teleosts. The *lepB* gene has been proposed to be involved in lipid metabolism in chub mackerel (253) and mandarin fish (289). However, plasma levels do not correlate with body adiposity in salmonids (293, 297). Leptin patterns in adipose tissue vary widely among species and between duplicates; in salmon, only *lepA1–2* are found with *lepA1* type being higher expressed (260, 261). Low *lepAI–II* expression has been reported in visceral adipose tissue of common carp (298). The differential leptin expression in adipose tissue between fish species and mammals may be a result of the divergent fat allocation patterns observed for the various species but also related to differences between endotherm and ectotherms.

*In vivo* recombinant LepA treatments suggest anti-adipogenic effects and stimulatory actions on fat metabolism in several teleosts (287, 299, 300). Consistently, LepA treatment *in vitro* stimulates lipolysis in rainbow trout adipocytes (284). In addition, *lepr*-deficient medaka exhibit increased visceral fat depots compared to wild types, which is consistent with the body composition of the leptin receptor-deficient db/db mice and Zucker obese rats (243, 301).

While these findings suggest that leptin is involved in mobilization of lipid stores in fish, emerging literature suggests that rather than a canonic “lipostat” signaling for adipostasis (as in mammals), leptin might be important in other metabolic processes. Recent fish studies suggest roles of leptin in glucose homeostasis (302–304) and in the coordination of energy metabolism and somatic growth (305). Leptin receptor-deficient zebrafish do not exhibit increased appetite or adiposity but display β-cell hyperplasia and increased levels of *insulin* mRNA and alterations in glucose homeostasis, suggesting that leptin might act as a glucostat rather than a lipostat in fish. In both rainbow trout (303) and tilapia (304), either peripheral or central treatment of homologous LepA induces hyperglycemia and glycogenolysis. In tilapia, lipase gene expression was not altered, suggesting the hormone is important in mobilizing glucose. Thus, the contradictory leptin data attained so far on gene expression, *in vivo* and *in vitro* recombinant leptin administrations or leptin plasma levels in response to different feeding status, suggest an independent evolution of leptin functions among teleosts. Species-specific responses among orthologs may reflect defined metabolic adaptations to the widely diverse fish life histories. Similarly, leptin duplicates may be under different selective processes and respond to modulation of nutritional status in a spatiotemporal specific manner.

### Other Tissues

In mammalian species, there is a range of other peripheral tissues that produce and release factors (peptides/cytokines) that affect appetite, such as the thyroid and pancreatic hormones.

#### Thyroid

The thyroid axis consists of hypothalamic TRH, pituitary thyrotropin (TSH), and thyroid hormones [thyroxin (T4) and tri-iodothyronine (T3)]. In mammals, the thyroid axis plays a significant role in energy expenditure, as it increase basal metabolic rate, control appetite, and food intake and regulate body weight (306, 307). The few studies that have targeted the role of the thyroid axis on fish feeding suggest a stimulatory effect. For instance, in goldfish, injections of either TRH or T4 increase feeding and locomotion (82, 308), and treatment with the antifouling agent tributyltin increases weight gain and food intake, as well as serum thyroid hormone levels (309). In Amur sturgeon (*Acipenser schrenckii*), low feeding rates result in low thyroid hormones serum levels (310). In both winter flounder (72) and goldfish (82), fasting induces increases in hypothalamic *trh* mRNA expression, further suggesting an orexigenic role.

#### Pancreas

The pancreas secretes mainly insulin and glucagon-related peptides, which have been shown to affect metabolism in fish (311). Plasma insulin and glucagon levels increase after feeding in fish; however, their specific role in the food intake regulation is largely unknown.

Complete isletectomy in the goby (*Gillichthys mirabilis*) results in hyperphagia (312), and in rainbow trout, intraperitoneal injections of insulin decrease food intake (313), suggesting an anorexigenic role for insulin in fish.

The vertebrate proglucagon (*Pg*) gene encodes three peptide hormones, namely, glucagon, glucagon-like peptide 1 (GLP-1), and glucagon-like peptide 2 (GLP-2) (314). In mammals, GLP-1 and GLP-2 are satiety signals, mainly produced by the GI-tract (315, 316). In fishes, the pancreas synthesizes glucagon and Glp-1, and the intestine releases glucagon, Glp-1, and Glp-2 (317). To date, the *pg* gene has also been isolated in several teleost species (314), and duplicate *pg* genes have been identified in all teleost species for which the genomic sequencing has been completed (318). Although, to our knowledge, there is no information on glucagon and Glp2, Glp-1 appears to act as an anorexigenic factor in fish. In channel catfish, central administration of GLP-1 has a potent inhibitory effect on feed intake, but peripheral injection showed only a weak or no effect on appetite (188, 319). On the other hand, peripheral GLP-1 injection strongly decreased feed intake in Coho salmon (242), suggesting that the peripheral (GI-tract) anorexigenic Glp-1 effects might be species-specific in fish. In rainbow trout, peripheral injections of Glp-1 increase plasma glucose levels, decrease hindbrain *npy* and *pomc* mRNA levels and increase hindbrain *cart* expression levels, suggesting that Glp-1 regulates not only food intake but also glucose homeostasis (320). Although mammalian GLP-1 inhibits gastric emptying (321), the function of Glp-1 on digestion (speed) is still unclear in fish.

## Selected Fish Adaptations in the Endocrine Regulation of Feeding

Owing to their large diversity, fishes display a wide range of interesting adaptations in the feeding biology and appetite to different environmental conditions and food availability. Research on these comparative aspects both with regards to evolution and function is still largely unexplored and only a few species, mainly with commercial interest, have been studied. Below, we provide some examples and discuss other adaptions that could be explored further.

### Long-term Seasonal Fasting (The Arctic Charr)

The anadromous (sea-migrating) life-strategy of Arctic charr (*Salvelinus alpinus*) is characterized by substantial seasonal changes in food intake, growth, and adiposity. In the wild, most of the annual growth and energy accumulation occurs because of an intense appetite burst during the short seawater residence in summer, whereas overwintering in freshwater is characterized by anorexia and depletion of energy reserves (322–325). The seasonal cycle in food intake and growth in this species seems to be a strictly genetically programmed process as captive offspring of Arctic charr exhibit pronounced seasonal changes in food intake and growth when held at constant temperature and given food in excess (326, 327). Because of the physiologically regulated seasonal feeding cycles, Arctic charr represent an interesting model for investigation of adaptive mechanisms underlying long-term regulation of appetite and energy homeostasis (328).

It has been suggested that the seasonal feeding cycle is regulated by a lipostatic mechanism (297, 328–330). Leptin, the principal regulator of the lipostatic mechanism in mammals (331), does not appear to be involved in signaling the large variations of adiposity in the Arctic charr (297). However, hepatic leptin production increases at the end of the winter fasting period (297), when fat mobilization and increased plasma glucose occurs (325). It is possible that leptin has a role in depressing metabolism during long-term seasonal fasting, when fat stores are depleted by the suppression of liver lipolytic pathways (292, 297). It is also possible that leptin is more important as a glucostat than an adipostat in Arctic charr, as suggested in zebrafish (302).

The role of Ghrl in controlling the seasonal variation in appetite of charr has also been explored. Stomach *ghrl* mRNA expression seems to be negatively correlated with feed intake and growth (332), supporting that Ghrl acts as an anorexigenic factor, as suggested in one study on rainbow trout (191). The expression levels of a range of putative central appetite-controlling genes in Arctic charr such as *pomc, cart, mc4r, agrp*, and *npy* were not correlated to its annual feeding cycle (333). Further studies are needed to understand how anadromous Arctic charr can maintain an anorexic state when overwintering despite the massive loss of fat reserves.

### Long-term Fasting Related with Reproduction (The Mouthbrooder)

Mouthbrooder fish hold their eggs in their mouth until their young are free-swimming. Several fish are classified as mouthbrooders, some being paternal (male holds eggs) and others maternal (most common). Eggs can be fertilized in the environment or in the female’s mouth (in the case of maternal brooding). Teleost mouthbrooder fish include cichlids (e.g., mbuna *Astatotilapia burtoni*) and tilapias such as *Oreochromis mossambicus* and *Oreochromis niloticus*, sea catfish (e.g., *Ariopsis felis*), cardinalfish (e.g., *Pterapogon kauderni*), and gouramis (e.g., dwarf gourami *Colisa lalia*). While guarding eggs, most mouthbrooders do not eat or feed less, often resulting in a weight decrease (334–338).

Very little is known about the endocrine mechanisms responsible for brooding-induced fasting. Fed mbuna females with large ovarian eggs (pre-spawning or spawning) have larger gonadotropin-releasing hormone (Gnrh1) neurons (339), which has also been observed in convict cichlid, *Amatitlania nigrofasciatus* (340) and higher mRNA expression levels of whole brain *gnrh1* (major Gnrh form involved in reproduction), than mouthbrooding females carrying eggs, which is reflected by higher gonadosomatic indexes and higher circulating levels of sex steroids (341). However, no significant differences are seen in *gnrh2*, in contrast with fasting-induced changes reported for other fish species [e.g., winter flounder (342) and Ya fish (343)]. Similarly, no differences are seen in *npy, pomc* or *mch* whole-brain expression, between mbuna holding eggs in their mouths and pre-spawning females (341). However, *orexin* increases in fasting mbuna females, which is consistent with its stimulatory role on feeding and inhibitory actions on spawning (66). The increase in *cck* is more surprising, as Cck is a satiety factor that is normally secreted when the GI-tract is full. This increase in *cck* might be a response to long-term fasting to attenuate hunger and prevent feeding by counteracting increases in orexigenic peptides such as orexin.

Interestingly, when comparing fed and fasted mouthbrooding females from which eggs/fry have been removed, no differences in brain expressions of appetite regulators (*npy, cck, orexin, pomc*, and *mch*) were seen (341), possibly because of changes in physiology and metabolism. However, as no information is available about the effects of fasting on appetite regulators for pre-spawning females or immature fish, it is difficult to draw definitive conclusions on the changes that lead to brooding-induced fasting.

### Long-term Fasting in Aquaculture (Trout and Salmon)

Like the above-mentioned Arctic charr, many other fish species, including rainbow trout and Atlantic salmon, tolerate long fasting periods. Rather than a genetically driven seasonal halt in feed intake as in charr, they adapt to long periods with low food availability in the wild. To better understand the potential role of various peptides in this process, plasma protein and/or gene expression levels of candidate appetite-regulating hormones and neuropeptides have been analyzed during variable periods of food deprivation in salmon and trout.

#### Leptin

The picture of leptin endocrinology dynamics in fish during fasting is not clear-cut, even within species, e.g., rainbow trout. Recent data on two lines of rainbow trout bred for either high (fat line) or low (lean line) muscle lipid content indicate that leptin response to fasting may be plastic and dependent on selective breeding, environmental factors and/or energy status and body composition (344). The two lines of trout differ in the fat deposition pattern: the fat line has higher total energy reserves, higher muscle adiposity, and lower visceral adiposity than the lean line. A 4-week fasting period decreased plasma Lep in the lean line while Lep levels and hepatic *lep* expression remained unchanged in the fat line (344). This contrasts previous results in rainbow trout, where leptin levels increase or remain unchanged during fasting, despite a decrease in condition factor (293, 345).

Tissue *lep* gene expression was also unaltered in long-term fasted fish except for an increased expression in fat rich muscle tissue (346). In the same study, the fasted fish displayed hyperphagia when they could refeed, eating as much as up to 8.4% of their body weight (346). Hence, even though the fasted fish were clearly in a catabolic state, hungry and mobilizing energy stores, leptin production and plasma levels remained unchanged.

Unlike the observation mentioned above (346), appetite does not always return immediately when food becomes available for anorectic/food-deprived salmonids (345, 347). During a 72-h refeeding period for long-term fasted rainbow trout, there was a large variability in the time to start feeding between individuals, and some did not feed at all in the beginning. This response may have been caused by high leptin levels in these individuals (345). Leptin generally did not start to decrease until some food had been ingested, raising the question of which mechanism is responsible for triggering the onset of appetite. In fine flounder (*Paralichthys adspersus*), leptin also decreases after, but not before refeeding (291). This fast leptin response indicates that there is a short-term meal-related regulation of leptin release (291, 345).

Available data on the relation between leptin and energy status in Atlantic salmon are still limited to those from food restriction studies or experiments using diets with different energy content (260, 290, 348, 349). Plasma leptin levels were not different between fish that were fed full or restricted (60%) rations for 10 months, although hepatic *lepA2* expression was higher in the fed than in the fasted salmon (260). In a shorter trial (7 weeks), feed-restricted fish had higher plasma leptin levels and elevated hepatic *lep* expression levels than controls fed to satiation (290), which is consistent with some of the previous studies on rainbow trout (293, 345). Restricted feeding during several months (April–September) in Atlantic salmon parr undergoing sexual maturation showed that fish with the highest fat stores had the lowest leptin levels (349). Similarly, fish on a high-energy diet had lower leptin levels than fish on a low energy diet with less adipose stores (348). Taken together, these studies lend further support to the notion that leptin is not a long-term adiposity signal in salmonids. The results obtained from fish species are also interesting in the context of studies on wild mammals with seasonal changes in adiposity and feeding behavior, showing a large variability in the link between plasma leptin levels, fasting, and adiposity (350–353).

#### Ghrelin

The response of plasma Ghrl and *ghrl* mRNA expression to fasting in fish is highly variable between studies and fasting duration (354). There are few studies investigating the response of Ghrl to long-term fasting in Atlantic salmon and rainbow trout. In rainbow trout, plasma Ghrl levels decreased after 1–3 weeks of fasting (213). In Atlantic salmon, 2 days of fasting led to elevated plasma Ghrl levels, indicating an effect of short-term feeding status on Ghrl release, a response consistent with this “hunger hormone.” However, after 14 days of food-deprivation, Ghrl levels were unchanged in fasted salmon compared to fully fed controls (355). Whether these differences are a result of true species differences in Ghrl function (see section above about ghrelin), domestication processes or experimental design remains unclear.

#### Fasting-Induced Changes in Central Appetite Regulatory Neuropeptides

The recent study by Jørgensen et al. (346) is one of few that have investigated potential changes in the expression of hypothalamic appetite-regulating peptides during fasting in a salmonid species. Rainbow trout was fasted for 4 months, and among the peptides that were measured in the hypothalamus (*lepa1, cart, agrp, pomca1, pomca2, pomcb, npy, mc4r*, and c*rf*), few fasting-induced effects were observed. There was an increased gene expression of *pomca1* and *pomcb*, suggesting that increased *pomc* transcript levels may be a potential mechanism for a reduced appetite and foraging activity in catabolic conditions.

Peripherally injected Lep seems to increase the expression of *pomc-a1* and *-a2* with a concurrent transient reduction in *npy* gene expression (279). In rainbow trout, the leptin receptor is localized in mediobasal hypothalamic appetite centers, and it seems that Pomc and Cart mediate leptin’s acute anorexigenic effect in this species (295). It may be speculated that during long-term fasting in salmonids, increased circulating leptin levels stimulate hypothalamic Pomc neurons, suppressing appetite. Brain sensitivity (amount of receptor levels) to, e.g., leptin and Ghrl will also influence appetite. At the termination of a 7-week feeding/fasting experiment, fed Atlantic salmon parr showed an increase in *lepr* gene expression in the brain, while the *lepr* gene expression in food-deprived fish was unaltered despite increased plasma Lep levels. This was interpreted by the authors as the possible result of a negative feedback of Lep on its receptor (290).

### Life-Stage Transition (First Feeding Larvae to Juveniles)

Most fish species spawn eggs, in which the developing embryo relies on yolk nutrients until it is sufficiently developed to capture, ingest, and digest feed. After onset of exogenous feeding, the larvae continue to grow and develop into juveniles—a transition triggered by environmental cues that induce a coordinated program to remodel the organism. The transition involves a wide range of changes in behavior, habitat, and physiology, and many fish larvae change food sources as they become adults; therefore, it has major consequences for feeding behavior and most likely in the control of appetite (356).

Several studies have aimed to understand the various aspects of the feeding biology and nutritional requirements of developing fish larvae to improve their performance in aquaculture. However, very few have focused on the mechanisms that control appetite and food intake (42, 357). This may be partly explained by biological and technical challenges when working with fish larvae, such as the accurate determination of food intake, the use of individual larva (instead of pools), or the handling of individual variability in growth and development.

There are several described cases where fish larvae continue to eat, despite having an apparently full GI-tract. For instance, Atlantic halibut larvae continue to ingest prey despite a full gut and with gut transit rates so high that the prey is eliminated (defecated) undigested and sometimes even alive (358). Apparently, the feedback systems and satiety signals originating in the GI-tract are not functional in these early stages. It has been argued that fish larvae have adapted to low concentrations and availability of prey in the wild. Consequently, satiety signals may not be required to prevent overfeeding. In aquaculture conditions, however, larvae are reared with constant and abundant food availability and continuous light, and therefore appetite-controlling mechanisms become crucial to avoid continuous ingestion of prey, short gut transit times of ingested food, reduced time for digestion, low digestive efficiency, and nutrient absorption (359). This is of particularly interest for altricial-gastric species, which lack a fully developed and functional stomach prior to metamorphosis (360–364).

Some studies have started to explore the ontogeny expression of several appetite regulators (71, 240, 365, 366), and their detailed spatial and differential distribution in fish larvae (159). Key factors in appetite control are present very early in fish development, such as *npy* at zygote stage in blunt snout bream (*Megalobrama amblycephala*) (367) and at blastula stages in orange-spotted grouper (170), *ghrl* (240) and *ox* (71) at cleavage stage, and *gastrin* (240) at blastula stage in Atlantic cod. In Atlantic halibut, only *ghrl* and *cart* mRNA expression levels were significantly modified throughout development, while ontogeny did not affect *npy, pyy*, and *pomc-c* expressions levels in the brain of the developing larvae (35). Ghrl was widely distributed in the GI-tract and present in the anterior GI-tract before the gastric glands and pepsinogen production appeared in newly Atlantic halibut hatched yolk-sac larvae (368). Notably, increased levels of *ghrl* in the GI-tract during metamorphosis were correlated with stomach development (360, 369). *cart* mRNA expression levels decreased at the initiation of halibut metamorphosis, while *cart* levels in whole larvae of Atlantic cod increased during the corresponding developmental phase (365). In Atlantic cod, *cck, npy*, and *ox* show a similar pattern of a moderate but consistent decrease from 3 days post-hatching (dph) until 60 dph (42, 365). The differences in *cart* expression between Atlantic halibut and Atlantic cod larvae are intriguing and may be a result of different factors, including the use of whole cod larvae versus halibut head and differences in developmental rate (370, 371).

Many of the neuropeptides involved in appetite control in higher vertebrates and adult teleost are present in the brain of fish larvae, suggesting a role of these genes in appetite control also in the early stages (35, 159, 168, 372–374). In the recent study of Le et al. (159), the development expression patterns of *npy, cart*, and *ox* genes were analyzed in brain regions of Atlantic cod, from start of exogenous feeding until juvenile stage. Both spatial and temporal expression patterns of orexigenic and anorexigenic factors during larval ontogeny indicated a progressive development of the brain regulatory networks that control appetite. In addition, the wide distribution and co-expression of *npy, cart*, and *ox* in hypothalamus, led the authors to propose that this is the main area for appetite control in fish larvae, comparable to mammals and adult fish (6, 374–376). However, it remains unclear to what extent these appetite-regulating genes are functional at these early developmental stages.

Few have assessed the response of these factors in terms of feed intake (35, 40) or different diets (40, 42, 377). In Atlantic cod larvae, Kortner et al. (42) showed that the expression levels of *cck* and *npy* were diet-specifically modulated and followed the same expression profile as the genes coding for digestive enzymes, suggesting a close connection between appetite control and digestion processes. Recently, two studies in Senegalese sole larvae have analyzed the effect of fatty acids ingestion in the control of food intake (378, 379). The administration of several fatty acids (leate, linoleate, α-linolenate, or eicosapentaenoate) in sole post-larvae enhanced the expression of the anorexigenic neuropeptides *cart4* and *pomcb* and decreased the orexigenic *npy*, with no major discrepancies between the different fatty acids tested (378). However, the transcriptional analysis of several anorexigenic: *pyya, pyyb, glp1, cckl, cart1a, cart1b, cart2a, cart4, pomc-a, pomc-b, crf*; and orexigenic: *gal, npy, agrp2* factors showed a dissimilar response to feeding times and dietary fatty acid composition (cod liver oil, linseed oil, soybean oil, or olive oil) that was generally not in agreement with their putative function (40). For example, the changes observed for sole *npy* in developmental stages 16 and 34 dph were not consistent. At 16 dph *npy* expression levels increased before feeding, as expected, but then continue to increase up to 3 h after feeding (40), which is counterintuitive for an orexigenic factor (1, 12). At 34 dph, *npy* expression was only affected by the dietary fatty acid profile. This was similar to the results obtained by Kortner et al. (42), where cod *npy* was diet-specifically modulated in larvae at 16 dph, but no evident changes were found at 29 dph. Furthermore, in Atlantic halibut larvae, *npy* levels increased 5 h after refeeding (35). The differences observed between species may suggest that the Npy is still not fully functional in appetite regulation in larvae, possibly reflecting a yet underdeveloped appetite-regulating system. Furthermore, the response of *npy, pyy, pomc-c*, and *cart* to food deprivation and refeeding in Atlantic halibut larvae did not appear to be coordinated (35), lacking a consistent expression pattern to explain their contribution to appetite control in early larvae as it was for Senegalese sole larvae (40). In addition, the differences observed between both studies in Senegalese sole larvae may be explained by the different approaches used: use of complex diets fed through the whole larval and post-larval stage (379) versus a tube-fed single meal of pure fatty acids solution (378).

Altogether, these studies support the hypothesis that a feedback signaling system from the GI-tract to the CNS is still not fully established in the early larval stages. This, however, does not rule out that developing fish larvae may have their own specific system of appetite regulation adapted to their feeding ecology or that larvae possess a rudimentary, still developing, regulatory system. Fish larvae are often considered as “feeding machines” because they can ingest food at rates above their own weight daily (357, 380–382). This suggests that larvae are constantly hungry and motivated to feed, although several studies have shown that some fish larvae exhibit a circadian prandial pattern and do not feed constantly (383–385). Given the complexity of appetite-controlling mechanisms and how difficult it is to interpret results due to the lack of specific information on the roles played by some of the potential anorexigenic and orexigenic factors in fish, it remains a challenge to elucidate the appetite-control system in fish larvae with different digestive tract morphologies and feeding strategies. A better understanding will greatly increase our basic knowledge on larval physiology and help to improve larval rearing regimes and feeding protocols in hatcheries.

### The Voracious Feeders

Several species have an aggressive and voracious feeding behavior, most of them usually being carnivorous top predators. Well-known examples include Perciformes such as bluefish (*Pomatomus saltatrix*), bluegill (*Lepomis macrochirus*), cobia, groupers, tilapia and African cichlids, salmonids (e.g., rainbow trout), pikes (e.g., Northern pike *Esox lucius*), some characids (e.g., dourado and piranhas), as well as elasmobranchs, i.e., sharks and rays (338).

Within the teleosts, several studies have examined the effects of fasting and feeding on the expression of a few appetite regulator genes. However, there are no data on how endocrine mechanisms might regulate the increased feed intake in these voracious fish, and no comparative study has been performed between voracious species and a “gentler” herbivore/omnivore species (e.g., cyprinids, some flatfish species).

In response to fasting, it appears that most voracious fish display a similar trend to what occurs in non-aggressive species [e.g., the omnivorous goldfish and pacu (*Piaractus mesopotamicus*)], i.e., increases in expression of orexigenic factors [e.g., *ox* in dourado (73) and piranha (225), and *ghrl* in piranha (386)] and decreases in expression of anorexigenic factors [e.g., *cart* in piranha (225)]. However, few studies have examined periprandial changes in voracious fish. Taking the example of orexin, its expression appears to increase around feeding time and decrease after feeding, similar to what is seen for other fish species, such as orange grouper (70) and tilapia (387). In dourado, *ox* expression is similar before, during, and after feeding, suggesting a constant state of feeding/searching behavior. In addition, *ox* expression levels in fasted fish increase at mealtime and dramatically at post-feeding time, suggesting that dourado have a high motivation to search for food that persists after meal time (73). In contrast, pacu, a fish from the same order (Characiformes) as dourado, shows high *ox* levels at pre-feeding, and these tend to decrease at mealtime and post-feeding. Moreover, if pacu is not fed at the scheduled mealtime, *ox* levels increase at mealtime but return to basal levels within 1 h, suggesting that the fish have “given up” on searching food (388), which is reflected by their calm behavior (Volkoff, personal observation).

Voracious fish are often aggressive during feeding. Although aggression is often related to reproduction, in these species it also occurs outside the reproductive context (389). Interestingly, early studies in cichlid fish (*Tilapia heudelotii macrocephala*) and in bluegill have shown that electrical stimulation of the hypothalamic region elicited both feeding and aggressive responses (390, 391). The brain monoaminergic system, especially serotonin [5-hydroxytryptamine (5-HT)], plays a key role in controlling aggressive behavior (392). 5-HT has been reported to inhibit aggressive behavior in several voracious species, e.g., trout (393) and pikeperch (*Sander lucioperca*) (394). Interestingly, surface Mexican tetra (*Astyanax mexicanus*) species are aggressive predators, in particular during feeding episodes, whereas blind cave forms of this species exhibit reduced aggressiveness and have a tendency to continuously search for food. These differences in foraging and aggressive behaviors are related to 5-HT network modifications within hypothalamic neurons (395, 396). 5-HT also has anorexigenic actions in rainbow trout (397) and in mammals (387) and has been shown to interact with appetite regulators. For example, the behavioral effects produced by orexin administration, i.e., increased locomotion and feeding, are blocked by 5-HT antagonists (398). It would therefore be valuable to compare 5-HT levels between voracious and non-voracious fish.

Intra-species differences (sometimes referred to as personality/motivation) in basal locomotor and feeding activities are often observed between individuals. These differences might be due to different expression levels of appetite regulators or monoamines. For example, in tilapia, low serotonergic activity in the hypothalamus is correlated with a personality characterized by high feeding motivation (399). Similarly, in salmonid fish, subordinate individuals characteristically exhibit higher plasma cortisol levels than dominant ones (400). There are most likely different causes for voraciousness in fish, and more direct studies are needed to explain the underlying mechanisms of the appetite-controlling networks that result in these large differences in feeding behaviors.

### How Important Is Vision? (The Blind Mexican Cavefish)

Although most fish rely in part on vision to feed (401), this sense is not essential for some species. The best example is that of fish living in cave environments, which are characterized by constant darkness and food scarcity (338, 402). Cavefish such as the Mexican tetra are often blind and have specialized anatomical features to better locate food and maximize food intake (396, 403, 404). Such adaptations include well-developed olfactory bulbs (405), taste buds (406), and lateral line neuromasts (407–409). In addition, these fish display behavioral adaptations for detecting prey and increasing feeding efficiency: they are opportunistic feeders, show increased swimming/exploratory and feeding behaviors (410), do not sleep (411), and do not exhibit schooling behavior (403, 412, 413). This enhanced food-finding efficiency is present not only in adults but also in young larvae when the yolk has been depleted (414). Overall, surface fish placed in the dark are less efficient at finding food than cavefish (415–417).

To cope with a particularly food-limited habitat compared to most surface fish, cavefish have developed behavioral (increased appetite, with ingestion of large amounts of food during feeding events) and metabolic adaptations. The latter include reduced basal metabolic rate, increased metabolic efficiency, starvation resistance (reduced weight loss during fasting), and increased body fat composition (403, 413, 418).

Peripheral injections of known orexigenic factors in cavefish, such as OX, GHRL, and apelin, increase not only food consumption but also the whole brain mRNA expressions of orexigenic factors (e.g., GHRL injections induce an increase in *ox* brain expression), whereas injections of CCK reduce food intake and induce a decrease in the whole brain expression orexigenic factors (e.g., *apelin*) (67, 79). Peripheral injections of OX greatly increase locomotor activity and *ox* brain mRNA levels in cavefish. Basal *ox* mRNA levels in whole brain are higher in cave fish than in surface fish (Buenos Aires tetra, *Hyphessobrycon anisitsi*, a characid surface species closely related to *Astyanax*) (405), suggesting that the higher overall locomotor/feeding activity in cavefish compared to the surface forms might be mediated by an increase in *ox* levels (67, 79). Coding mutations in *mc4r* also contribute to the increased appetite and starvation resistance of cavefish compared with surface fish (419).

Cavefish are avid feeders and become very active around feeding time when appetite increases (420). Brain *ox* mRNA expression levels increase before and decrease after a scheduled mealtime (67), suggesting that orexin acts as a short-term hunger signal and is linked to food anticipatory activity. Conversely, the brain expression of the anorexigenic *pyy* increases after feeding (67), suggesting a role for Pyy as a short-term satiety factor. However, *cck* brain expression does not display periprandial variations in cavefish (67), which might contribute to a less rapid satiety and longer bouts of feeding.

Short-term food restriction increases *ox* brain mRNA transcription levels in cavefish (67), indicating a role in the long-term regulation of feeding in cavefish and perhaps triggering an increased motivation to seek food. However, as opposed to most surface fish examined to date, short-term fasting does not increase brain mRNA levels of *pyy* and *cck*, suggesting that the anorexigenic systems are inhibited during fasting, perhaps to slow down digestion/gastric emptying of food in the gut or to maintain a hunger state that would favor food-seeking behavior.

## Future

Many of the studies on appetite-controlling systems in teleosts are based on domesticated fish that have been bred in captivity for generations (e.g., salmon, carp, and cod). These fish, which are submitted to optimal habitat (e.g., no predators, constant optimal photoperiods and temperatures) and feeding (e.g., satiation, minimal food-seeking behavior) conditions might have present modifications in their feeding behavior and systems controlling appetite, as compared to wild fish exposed to suboptimal conditions. This phenomenon has been shown in domesticated rats that eat more than wild individuals (421). Comparisons between wild and captive populations might reveal important information on the effects of domestication on feeding behavior. Therefore, observations of feeding behavior and sampling of fish in their natural environment would be valuable.

Overall, within a few model species, only a few appetite-regulating hormones (e.g., leptin, Npy, and Cck) have been studied more in detail. In addition, there are very few studies on the mechanisms of action of these hormones, including at the level of their target cells and their receptors. Many questions related to the concepts “set-point” in energy homeostasis and stimulus for synthesis/secretion of these hormones, i.e., whether it is direct nutrient sensing by the hormone-producing cells or stimulation of these cells by another hormone/neurotransmitter or both, also remain to be answered. Also, many of these hormones are expressed both in the CNS and in peripheral tissues and the relative importance of each, as well as their interactions in controlling the appetite, are poorly understood.

One of the major limitations in the field of appetite endocrinology in fish is that the vast majority of studies have been constrained to the analyses of transcript levels. Although the existence of a proportional relationship between mRNA and protein expressions measured from a tissue have long been assumed, recent data show that this is not always the case (422). The development of fish-specific hormone assays and protein expression techniques is crucial for a better understanding of appetite-regulating mechanisms in fish. In addition, most studies analyze large portions of specific tissues (e.g., whole brain, whole hypothalamus, or whole intestine), which might also bias results, as, for example, specific regions (e.g., proximal versus distal intestine, or specific hypothalamic nuclei) might have different functions and respond differently to feeding conditions.

Although it is often observed that growth is directly related to food intake, many gaps exist on our understanding of how these two functions are connected in fish. The recent development of GH-transgenic fish is promising for the exploration of this field. Thus, the development of emerging techniques such as gene editing (CRISPR/Cas9 system) will be a great tool to study the role of appetite regulators in fish. Targeted mutagenesis using CRISPR/Cas9 system has been successfully used in several species, including zebrafish (423), salmon (424), and African cichlids (425), but so far only a few studies have used this technique to examine the role of appetite regulators on fish models, e.g., leptin receptor mutations in zebrafish (302).

## Author Contributions

All authors designed, wrote and approved the final version of the manuscript.

## Conflict of Interest Statement

The authors declare that the research was conducted in the absence of any commercial or financial relationships that could be construed as a potential conflict of interest.

## Terminology for Gene Names

GENE (All capitals), Mammalian protein; Gene (First letter capital), Fish Protein; *Gene* (First letter capital, italic), Mammalian gene; *gene* (small letters only and italic), Fish gene.
